# The hospital resource utilization associated with osteoporotic hip fractures in Kermanshah, Iran

**DOI:** 10.5249/jivr.v6i1.492

**Published:** 2014-01

**Authors:** Morteza Saeb, Mandana Beyranvand, Zahra Basiri, Hassan Haghparast-Bidgoli

**Affiliations:** ^*a*^Department of Orthopedic Surgery, Imam Reza Hospital, Kermanshah University of Medical Science, Kermanshah, Iran.; ^*b*^Department of Internal Medicine, School of Medicine, AJA University of Medical Science, Tehran, Iran.; ^*c*^Department of Internal Medicine, School of Medicine, Hamedan University of Medical Science, Hamedan, Iran.; ^*d*^University College London, Institute for Global Health, London, UK.

**Keywords:** Hospitalization costs, Hospital length of stay, Hip fracture, Osteoporosis, Iran

## Abstract

**Background:**

Hip fracture is the most serious complication of osteoporosis and imposes a significant financial burden on countries' economy. This study aimed to assess the hospitalization costs and length of stay associated with osteoporosis hip fractures and identify the major cost components in a referral hospital in Kermanshah city, Iran.

**Methods:**

In a prospective study, from May 21 2007 to May 21 2008, all patients with osteoporotic hip fracture admitted to a referral hospital for operation were recruited as the study sample. For each patient, information such as age, gender, length of stay (LOS) in hospital and intensive care unit (ICU), medical and diagnostic procedures and cost of surgery and implant were collected both through interview with the patient or a family member and the patients’ hospital records.

**Results:**

A total of 103 patients (56 men and 47 women) were studied. The average hospital length of stay (LOS) for the patients was 9.7 days, ranging from 5 to 38 days. The average total hospitalization costs was 7,208,588 IRR (US$774). The main components of the costs were ward stay (16.3%), operative (54.6%), implant (26%) and medical and diagnostic procedures (3.1%).

**Conclusions:**

The results of this study demonstrate that the hospital resource burden associated with osteoporotic hip fractures in Iran is substantial and expected to rise with the projected increase of life expectancy and the number of elderly in Iran. Estimating the economic burden of osteoporotic hip fractures provide information that can be of importance in the planning and design of preventive strategies.

## Introduction

Osteoporosis is the most common metabolic bone disease worldwide, in which the bone mass decreases due to an imbalance in bone formation and resorption. It is associated with deterioration in skeletal micro architecture, which in turn increases the risk of bone fracture.^1-3^ Among all bone fractures, hip fractures are the most serious complication of osteoporosis and are shown to be responsible for substantial costs associated with hospitalization, surgery, outpatient care, long-term care, disability, and premature death which impose a major economic burden on patients, their families and the society in many countries.^4-6^ With aging of population and improving life expectancy worldwide, the incidence rate and financial burden of hip fractures will be rising.^1-3,7^

In Iran, where epidemiological information on osteoporotic hip fractures has been well documented in previous studies,^8-15^ there is little data reported on costs and resource utilization associated with osteoporotic hip fractures. Very few studies have previously tried to assess the economic burden of hip fractures in Iran.^16,17^ The current study aimed to assess the hospitalization costs and length of stay associated with osteoporotic hip fractures and identify the major cost components in a referral hospital in Kermanshah city.

## Methods

Kermanshah, the capital city of Kermanshah province, is located in the west of Iran. In Kermanshah city, there are six hospitals with orthopedic facilities (among them 5 are governmental facilities and 1 private sector).The main referral hospital in the city is Taleghani hospital. In a prospective study, from May 21 2007 to May 21 2008, all patients with osteoporotic hip fracture admitted in Taleghani hospital for operation recruited as sample. Hip fracture is defined as clinical and radiological evidence of fracture of the proximal femur (ICD-10: S72.0). For each patient, information such as age, gender, length of stay (LOS) in hospital and intensive care unit (ICU), medical and diagnostic procedures and costs of surgery and implant were collected both through interview with the patient or a family member and the patients’ hospital records. 

According to the patients’ medical history, data of discharged sheets and X-ray results, those patients who had car accidents or pathologic fractures were excluded from the study sample. During the two-year study period, 116 patients with osteoporotic hip fracture were admitted to the hospital. Among these patients, non-surgical cases were also excluded from the study and the remaining cases (i.e.103 patients) were considered as the final sample. The costs of medical treatment were divided into four major components: ward stay costs, operative (surgical) costs, implant costs and cost of medical and diagnostic procedures performed. The ward stay costs were included the costs of stay in Emergency Department (ED), orthopedic ward and Intensive Care Unit (ICU). The operative costs were included operating room costs, surgeons’ and anesthetists' fees. The therapeutical, surgical and all diagnostic procedures (e.g. lab tests, X-rays, ECG etc.) performed for each patient were carefully investigated and verified using the patients’ hospital records. After identifying and measuring resource used by the patients, the mean total hospitalization costs for patients was calculated. 

This study was approved by the ethical committees of Kermanshah University of Medical Science.

## Results

A total of 103 new cases (56 men and 47 women) of osteoporotic hip fracture met the inclusion criteria. The average age of the patients was 72 years-old (ranging from 50 to 91 years-old). In general, 87% of the patients were transported to the hospital from their homes either by ambulance or commercial vehicles and 12.6% referred from other governmental healthcare facilities.

The mortality rate during hospitalization was 10.7% (11 cases: 7 male and 4 females). Surgical operations were included 64 dynamic hip screws and 39 hemiarthroplasties. Twelve patients (11.6%) were admitted to ICU during hospitalization.

The average hospitalization costs for the patients was 7, 208,588 Iranian Rials (IRR) (US$774). The average LOS for the patients was 9.7 days, ranging from 5 to 38.

Table 1 presents the details of hospitalization costs according to its various components. Surgical costs contributed to 55% of the total hospitalization costs followed by the implant costs with 26%.

**Table 1:** Breakdown of the hospitalization costs

Item	Costs US$ (IRR)*	Proportion of total cost (%)
Ward stay	13,017 (121,320,816)	16.3
Operative/surgical	43,502 (405,433,956)	54.6
Medical and diagnostic procedures	2,475 (23,068,292)	3.1
Implant	20,672 (192,661,500)	26
Total costs	79,666 (742,484,564)	100
Average costs	772 (7,208,588)	

*Conversion rates: US$ 1= 9320 IRR at 2007

## Discussion

There is little data reported on costs and resource utilization associated with osteoporotic hip fractures in Iran. The current study assessed the total hospitalization costs and LOS associated with osteoporotic hip fractures and identified the main cost components in a referral hospital in Kermanshah city. The average hospitalization costs and the average LOS for the patients in this study were US$774 and 9.7 days, respectively. 

Most studies measuring the costs of hip fractures have focused on the costs of the acute care. The costs of initial hospitalization have been reported vary significantly across different countries (Table 2). This variation can be explained by the difference in the time scale of different studies, incidence rate of osteoporotic hip fracture, effect of inflation rate and variation in organization of acute care service in various countries. 

**Table 2:** The comparison of the average costs and LOS associated with hip fractures across various settings.

Study	Year of study	Patients (N)	Average LOS (days)	Hospitalization costs(US$)	Country
Present study	2007	103	9.8	774(7,208,588 IRR)	Iran
Zamani et al ^16^	2005-7	119	7.9	305(2,844,464 IRR)	Iran
Navab et al^[Bibr B17]17^	2003-6	164	8	830 (7,735,600 IRR)	Iran
Lawrence et al^4^	2003	100	23	24,647(12,163 GBP)	UK
Dhar^19^	2005	16	16	2,627(1,010.6 OR)	Oman
Bubshait et al^6^	2006	43	17.7	13,019( 48,712 SAR)	Saudi Arabia
Lee et al^18^	2001	62	16	7,014(10,515 SGD)	Singapore
Clark et al^20^	2005	218	10.7	3,921.1	Mexico
Wiktorowicz et al ^5^	1995-6	541	21	9,804 (9,820 CAD)	Canada
Autier et al^21^	1995-6	170	29	12,229(8,667 EUR )	Belgium
Levy et al^22^	1999	12141	-	8,325(5,900 EUR)	France

The estimated hospitalization costs and LOS in the current study are similar to the previous studies conducted in Iran. ^16,17^ However, the average costs and LOS reported in the current study and the previous Iranian studies are significantly lower compared with the average costs and LOS reported in the studies from other settings(Table 2).

The results of studies from various countries suggest that the osteoporotic hip fractures impose a high financial burden on the societies, which is mostly associated with the duration of hospital stay.^4,18,19,6,20,5,21,22^

In a study by Lawrence et al, ward stay costs contributed to 84% of the total osteoporotic hip fracture costs. They reported that 40% of patients experienced a delay before undergoing surgery and the majority of hospitalization days were spent for recovery after surgery.^4^ Furthermore, Lee et al in their study found that the patient's LOS had a significant positive correlation with the hospitalization costs.^18^ As it has been shown in Table II, the average LOS for the patients in the current study and the previous studies in Iran were lower compared to the studies from other countries, which might explain to some extent the lower costs for osteoporotic hip fractures in Iran.

A further explanation for the lower costs of osteoporotic hip fractures in Iran compared with other countries, can be the subsidized health care system and also the general low service costs in Iran. The operation costs in our study, for example, were lower than the costs in most countries. For better understanding, we have compared the major components of hospitalization costs including the surgical costs, implant and hospital or ward stay costs in the studies from the selected countries, shown in Table 3.

**Table 3:** Comparing the average costs of operative, implant and ward stay between selected studies.

Study	Operative and implant costs٭ (US$)	%	Ward stay costs (US$)	%
Present study	623(3,936,252+1,870,500 IRR)٭٭	81	126.4(1,177,872 IRR)	16
Lawrence et al ^4^	2,298.8(1,134.4 GBP)٭٭	9	20,701.7(10,216 GBP)	84
Dhar^19^	551(212 OR )٭٭	21	1,576.8(606.7 OR)	60
Bubshait et al ^6^	3,446(11,954+942 SAR)	26	7,085.7(26,511 SAR)	54
Clark et al ^20^	1,771.1(1,110.6+660.5)	45	1,893	48

* In few studies ^4,19^ the costs of implants were included in the operative costs, therefore we summed up the operative and implant costs in other studies for better comparison. **The average costs of operative and ward stay was calculated as total costs of the items divided by total number of the patients.

Previous studies in Iran have shown that the incidence of osteoporotic hip fracture in Iran was lower than the developed countries.^12,13,23,15^ For instance, Ahmadi-Abhari et al.^15^ in their study of measuring the burden of hip fracture in Iran have concluded that the osteoporotic hip fracture does not contribute significantly to the disease burden in the country, considering its low incidence. However, as the life expectancy for the Iranian population is increasing,^24^ the rise of financial burden of hip fracture will be anticipated. 

We acknowledge a few limitations with the current study. This study only included hospitalization (inpatient) costs associated to osteoporotic hip fractures, and other direct medical costs such as rehabilitation and outpatient visit costs were not measured. Moreover, the hospitalization costs in this study are charged billed by the hospital and may not reflect actual payments nor true hospital costs due to factors such as government subsidies to hospital services and medicines, discounts and exemptions given to the patients and also not including out-of-pocket payments by the patients. Furthermore, the non-medical direct costs such as travel costs and the indirect costs related to informal care, as well as the costs associated with the pain and suffering by the patients and their families are not included in this study. Measuring these costs was beyond the scope of this study. 

## Conclusion

The results of this study demonstrate that the hospital resource burden associated with osteoporotic hip fractures in Iran is high. It is expected that the costs of osteoporotic hip fractures to rise with the projected increase of life expectancy and the number of elderly in Iran. This study only assessed the hospitalization costs of osteoporotic hip fractures which are only a small part of the costs. Further studies are required to investigate the full economic burden of osteoporotic hip fractures in Iran. Estimating the economic burden of osteoporotic hip fractures provide information that can be of importance in the planning and design of preventive strategies.
